# The reanimation of pseudoscience in machine learning and its ethical repercussions

**DOI:** 10.1016/j.patter.2024.101027

**Published:** 2024-08-01

**Authors:** Mel Andrews, Andrew Smart, Abeba Birhane

**Affiliations:** 1 The University of Cincinnati, Cincinnati, OH, USA; 2 Carnegie Mellon University, Pittsburgh, PA, USA; 3 Google Research, San Francisco, CA, USA; 4 Trinity College Dublin, Dublin, Ireland; 5 Mozilla Foundation, San Francisco, CA, USA

**Keywords:** machine learning, deep learning, AI ethics, physiognomy, philosophy of science, pseudoscience, epistemology

## Abstract

The present perspective outlines how epistemically baseless and ethically pernicious paradigms are recycled back into the scientific literature via machine learning (ML) and explores connections between these two dimensions of failure. We hold up the renewed emergence of physiognomic methods, facilitated by ML, as a case study in the harmful repercussions of ML-laundered junk science. A summary and analysis of several such studies is delivered, with attention to the means by which unsound research lends itself to social harms. We explore some of the many factors contributing to poor practice in applied ML. In conclusion, we offer resources for research best practices to developers and practitioners.

## The bigger picture

Machine learning has a pseudoscience problem. An abundance of ethical issues arising from the use of machine learning (ML)-based technologies—by now, well documented—is inextricably entwined with the systematic epistemic misuse of these tools. We take a recent resurgence of deep learning-assisted physiognomic research as a case study in the relationship between ML-based pseudoscience and attendant social harms—the standard purview of “AI ethics.” In practice, the epistemic and ethical dimensions of ML misuse often arise from shared underlying reasons and are resolvable by the same pathways. Recent use of ML toward the ends of predicting protected attributes from photographs highlights the need for philosophical, historical, and domain-specific perspectives of particular sciences in the prevention and remediation of misused ML.

## Teaser

Machine learning has a pseudoscience problem. In this perspective, the authors explore the recent resurgence of deep learning-assisted physiognomy and argue that pseudoscientific and socially harmful applications of machine learning often arise from shared underlying epistemic failings. They urge researchers to reject notions that machine learning can be theory free and to consider their work in an appropriate social and historical context.

## Introduction

The fields of AI/machine learning (ML) ethics and responsible AI have documented an abundance of social harms enabled by the methods of ML, both actual and potential. Although the topic is comparatively more obscure, critics have also sought to draw attention to the epistemic failings of ML-based systems: failures of functionality and scientific legitimacy.^1^^,^^2^ The connection between the ethicality and epistemic soundness of deployed ML, however, has received scant attention.^3^^,^^4^

We urge that if the field of AI ethics is to be efficacious in preventing and remediating the social harms flowing from deployed ML systems, it must first grapple with discrepancies between the presumed epistemic operation of these tools and their in-practice ability to achieve those aims. While such an observation is not novel (see Raji et al.^3^), we build on prior work, both in offering an analysis of the issue from a philosophical vantage point and in venturing into the intricacies of in-practice epistemic and ethical misuses of ML systems. We argue that philosophical, historical, and scientific perspectives are necessary in confronting these issues and that ethical and epistemic issues cannot, and should not, be confronted independently.

A recent surge of deep learning-based studies have claimed the ability to predict unobservable latent character traits, including homosexuality, political ideology, and criminality, from photographs of human faces or other records of outward appearance, including Alam et al.,^5^ Chandraprabha et al.,^6^ Hashemi and Hall,^7^ Kabir et al.,^8^ Kachur et al.,^9^ Kosinski et al.,^10^ Mindoro et al.,^11^ Parde et al.,^12^ Peterson et al.,^13^ Mujeeb Rahman and Subashini,^14^ Reece and Danforth,^15^ Su et al.,^16^ Tsuchiya et al.,^17^ Verma et al.,^18^ Vrskova et al.,^19^ and Wang and Kosinski.^20^,^21^ In response, government and industry actors have adapted such methods into technologies deployed on the public in the form of products such as Faception,^22^ Hirevue,^23^ and Turnitin.^24^ The term of art for methods endeavoring to predict character traits from human morphology is “physiognomy.” Research in the physiognomic tradition goes back centuries, and while the methods largely fell out of favor with the downfall of the Third Reich, the prospects of ML have renewed scientific interest in the subject. Much like historical forays into this domain, this new wave of physiognomy, resurrected and yet not, apparently, sufficiently rebranded, has faced harsh criticism on both ethical and epistemic grounds.^25^^,^^26^^,^^27^^,^^28^

This critical response, however, has yet to explore how the confused inferential bases of these studies are responsible for their ethically problematic nature. There are several conclusions we wish to draw from the detailed study of these examples, which we believe extrapolate to the relation between ethical and epistemic issues in deployments of ML at large.

(1) No inference is theory neutral.^29^
(2) Leaving a theory or hypothesis tacit means it is not held to account for, and its conclusions are not critically evaluated before the results of such work are deployed or acted upon.
(3) If a study informs a policy, intervention, or technology that will materially impact human lives—in other words, if a study is at all informative—and it misrepresents the human reality within which it is being deployed, it should be expected that harms to humans will arise. Wrong theories generate wrong interventions. Wrong interventions cause harm.
(4) ML models are developed and deployed to extract complex, high-dimensional statistical patterns from large datasets. These complex patterns are typically taken to represent unobservable latent features of the systems from which their training data were drawn. The norms and procedures established for correctly inferring unobservable latent variables from correlational measures differ by scientific field and must be indexed to subject matter.
(5) Meta-narratives and cycles of hype surrounding ML, we argue, play a direct role in encouraging errant usage of the tools. When ML tools are proclaimed to deliver false inferences, the outcomes are rarely ethically innocuous. This is true in general but is all the more salient for ML tools deployed in socially sensitive arenas.

In bringing to light the connection between pseudoscientific methods in applied ML and the ethical harms they perpetuate, we hope to encourage greater care in the design and usage of such systems.

## Physiognomy resurrected

“Physiognomy” is “the facility to identify, from the form and constitution of external parts of the human body, chiefly the face, exclusive of all temporary signs of emotions, the constitution of the mind and the heart.”

Georg Christoph Lichtenberg, 1778

Recent years have seen an abundance of papers promulgating physiognomic methods resting on ML models.^26^ Work of this ilk is undertaken by academic research groups, private firms, and government agencies. A number of representative instances of each claim to have trained ML classifiers to predict personality, behavioral, or identity characteristics from image, text, voice, or other biometric data. Inferred labels have included race,^30^ sexuality,^20^,^21^ mental illness,^16^ criminal propensity,^7^ autism,^8^ and neuroticism.^9^

These studies have predominantly relied on deep learning neural networks (DNNs), sometimes in tandem with more simplistic regression techniques. The practice of wielding the methods of ML toward the (putative) prediction of internal mental states, dispositions, or behavioral propensities based on outwardly visible morphology has been labeled “AI pseudoscience,” “digital phrenology,” “physiognomic AI,” “AI snake oil,” “bogus AI,” and “junk science.”^31^ These technologies, however, do not only exist in the abstract—a growing number of companies now market physiognomic capabilities, including the ability to detect academic dishonesty in students^24^ and future performance in prospective employees.^23^ Remarkably, a single tool marketed to defense contractors boasts of the ability to predict “pedophilia,” “terrorism,” and “bingo playing.”^22^

In this section, we review the details of several representative examples of physiognomic ML. These case studies are intended to be illustrative of the kinds of reasoning, epistemic foundations, and logic behind research and applications of automated inference from images portraying human likenesses. The studies presented here are intended to be representative of the genre and not a comprehensive overview.

### Inferring sexual orientation

Utilizing DNNs,^20^,^21^ Wang and Kosinski extract features from images of human faces, which they then regress in a supervised learning task against self-reported sexual orientation labels. The classifier achieved 81% and 71% accuracy scores on sexual orientation for male and female subjects, respectively. These findings represent a higher classification accuracy than experimentally determined human judgment. The researchers scraped their data from social media profiles, claiming that training their classifiers on “self-taken, easily accessible digital facial images increases the ecological validity of our results.”^20^,^21^ Wang and Kosinski report that the “findings advance our understanding of the origins of sexual orientation.”^20^,^21^ The authors of the study explain the ability of their models to discriminate sexual orientation with the claim that “the faces of gay men and lesbians tend to be gender atypical.”^20^,^21^ The validation of this hypothesis depended on the training of an additional DNN for gender discrimination. This classifier assigned a likelihood to each face image of being female. The researchers then interpreted this likelihood as a measure of facial femininity, assessing the faces of homosexual-tagged individuals against an average femininity score for heterosexual individuals. The researchers claimed that their results revealed that “the faces of gay men were more feminine and the faces of lesbians were more masculine than those of their respective heterosexual counterparts.”^20^,^21^ “The high accuracy of the classifier,” Wang and Kosinski report, “confirmed that much of the information about sexual orientation is retained in fixed facial features.”^20^,^21^ The contention of the researchers is that high classification accuracy of sexual orientation from facial features, alongside the evidence they supply for the gender-atypicality of facial morphology, lends support for a particular theory of the genesis of same-sex attraction. The proposed hypothesis is the prenatal hormone theory (PHT) of homosexuality, which proposes that same-sex attraction is a developmental response to atypical testosterone exposure in fetal development. Wang and Kosinski’s results, they claim in their preprint, “provide strong support for the PHT, which argues that same-gender sexual orientation stems from the underexposure of male fetuses and overexposure of female fetuses to prenatal androgens responsible for the sexual differentiation of faces, preferences, and behavior.”^20^,^21^

### Personality psychology

Kachur et al.^9^ write that “morphological and social cues in a human face provide signals of human personality and behaviour.” Their stated hypothesis is that a “photograph contains cues about personality that can be extracted using machine learning.” The authors further claim to have “circumvented the reliability limitations of human raters by developing a neural network and training it on a large dataset labelled with self-reported Big Five traits.” Here, deep learning is invoked as a means to obtain objectivity beyond human judgment; however, the training dataset was self-labeled by human raters. The predictive accuracy is interpreted as *prima facie* evidence for their hypothesis that structural features of human faces contain information of human personality and behavior, and the authors state that their “study presents new evidence confirming that human personality is related to individual facial appearance.”^9^

In this study, participants self-reported personality characteristics by completing an online questionnaire and then uploaded several photographs, which the researchers then used to construct their training and test datasets. In this example, as in Wang and Kosinski,^20^,^21^ researchers used the accuracy of their ML model as confirmatory evidence of a joint causal basis for both facial morphology and self-reported personality. Kachur et al. report “several theoretical reasons to expect associations between facial images and personality” including that “genetic background contributes to both face and personality.”^9^ Kachur et al. described their results as being indicative of “a potential biological basis” to the discovered association between face images and self-reported personality characteristics.^9^

### “Abnormality” classification

A recent study constructed a “normal” and “abnormal” human facial expression dataset for the purpose of automatically detecting such abnormal traits as drug addiction, autism, and criminality from facial images.^8^ The authors argued that “facial expression reflects our mental activities and provides useful information on human behaviors.” Kabir et al. “developed a combined method of Convolutional Neural Network (CNN) and Recurrent Neural Network (RNN) to classify human abnormalities.” “This approach,” they contend “analyzes the human face and finds the abnormalities, such as Drug addiction, Autism, Criminalism [*sic*].”

The researchers utilized images “gathered from the web using the web gathering technique,” although the details of this technique were not further elucidated. It is not made clear within the scope of the manuscript on what basis images were classified as “normal,” “drug addicted,” “autistic,” or “criminal.” The researchers reported a validation accuracy of 89.5% on the four categories. The provenance of the labels is left undisclosed in this study, as are the validation criteria.

In a similar vein, Vrskova et al.^19^ claim to be able to diagnose “abnormal” human activities such as “begging,” “drunkenness,” “robbery,” and “terrorism” from video footage.

### Lie detection

Automated deception detection has long been of interest to law enforcement, judicial systems, academic institutions, corporations, and governments.^32^ A recent study by Tsuchiya et al.^17^ utilized facial analysis and ML toward the putative automatic detection of deception for remote job-interview scenarios. The stated purpose of this research was to create an ML-based tool to detect when someone on video call might be lying. Participants in this study were asked to knowingly generate false descriptions of images while being recorded via video and biometric sensors. The researchers then used these data to train an ML model to predict deception-based facial or head movements, pulse rate, or eye movements. The researchers obtained a high accuracy rate using their classifier on the four participants used in the study. As in the other studies reviewed here, the predictive accuracy of the model was taken to substantiate the hypothesis that particular facial features or movements are evidence of unobservable character or behavioral traits—in this instance, deception.

### Criminality detection

A study by Wu and Zhang^33^ purported to “empirically establish the validity of automated face-induced inference on criminality.” The authors trained four canonical ML models on a dataset of ID photographs of Chinese citizens to predict the label of criminality. Wu and Zhang stated that their models detect “criminality based solely on still face images, which is free of any biases of subjective judgments of human observers.”^33^ The convolutional neural network achieved an accuracy rate of 89.51% at picking out subjects who had been arrested for a crime. Hashemi and Hall^7^ claim to have also developed a deep learning-based criminality detector.

## A history of misbegotten science

Everything that is within can be known by what is without.

Paracelsus, ca 1534

The practice of physiognomy dates back to at least ancient Mesopotamia^34^ and has resurfaced in variable form in nearly every century since. Physiognomy aims to infer unobservable latent personality or behavioral characteristics from the outwardly visible human phenotype. The Babylonians spoke of “physiognomic omens”: physical features of the face believed to be predictive of life trajectory.^35^ Aristotle was himself a practitioner and proponent of physiognomic methods.^36^ Prominent British Enlightenment rationalist Robert Southey examined slaves held for sale in Lisbon “with a physiognomic eye, to see if they differed from the rest of the people.”^37^

In the 18th century, the Swiss physiognomist Johann Kaspar Lavater forwarded a theory of physiognomy based on intuitive judgments, claiming physical attractiveness to be revelatory of moral character: “In proportion as he is morally good, he is handsome; and ugly, in proportion as he is morally bad.”^38^ The late 19th century Italian physiognomist Casare Lombroso proposed a theory of the “born criminal type,” which could be discerned through analysis of facial structure.^39^ These research traditions persisted on into the 20th century. William Herbert Sheldon (1940) advanced a theory of “somatotypes,” a taxonomy of body shapes that were taken as indicators of intelligence, personality, and moral character. Based on this taxonomy, he claimed to have developed “scientific” predictors of criminality, leadership, and similar characteristics dependent upon his somatotypes.^40^

Throughout their long history, the development and refinement of the study of physiognomy, alongside phrenology and eugenicist research programs, were undertaken largely in support of creating or upholding systems of social stratification. Theories of eugenics, phrenology, and physiognomy rationalized institutionalized racial hierarchies and gave settler colonial and imperialist projects a benevolent veneer. Physiognomic theories served as a justification for the capture, exploitation, and dispossession of many human ethnic groups throughout the 19th century. Physiognomic reasoning served a critical role in legitimizing and legalizing chattel slavery, forced sterilization, and genocide. Harry Laughlin’s^41^ Model Eugenical Sterilization Law, for instance, was enshrined into United States legislature outlawing reproduction among individuals with “undesirable” characteristics.^42^ This set a legal precedent, which was then mimicked by the racial purity laws of the Third Reich. By the end of the Nazi regime, over 400,000 people had been sterilized against their will in conformity with this legal doctrine.^43^ With the downfall of the Nazi regime, the public lost its taste for eugenicist and physiognomic methods and policies built thereon. This did not altogether stop key proponents from persisting in these schools of thought, however. In 1960, the former Nazi scientist Otmar von Verschuer and the British eugenicist Roger Pearson founded the journal *Mankind Quarterly*, which sustained the publication and dissemination of eugenics research.^44^

Although physiognomy endured within mainstream science and philosophy for centuries, the methods were decried as unscientific even in their recurrent heydays. Pliny the Elder, a first-century philosopher, found it concerning that someone so evidently learned as Aristotle deigned to endorse the methods of physiognomy.^45^ The ancient Greek physician Galen, whose work had a profound influence on medical science from the 2nd century until the 17th, criticized physiognomic methods:

Physiognomists do not attempt to make an absolute statement about all the features; they too have learned from experience. If someone is very hairy on the chest, they declare him to be “high-spirited” (irascible), while if on the thighs, “lecherous.” But they still do not add the cause. . . . They have stated what has occurred, having left the cause of this untold. But the man who is a natural scientist attempts to discover, as of all other things, so too the causes of these.^46^

Here, Galen draws a distinction between the physiognomist and the scientist on the grounds that science attempts to uncover underlying causal mechanisms. Physiognomists, meanwhile, merely string together observations. Some of history’s greatest scientific discoveries have seemingly occurred on the basis of mere observational data, however. Sir Isaac Newton could hardly experimentally intervene on the solar system to learn the laws of gravitation. What makes observational data suffice for some scientific inferences, while, in others, it appears woefully inadequate?

An 18th century critic of physiognomic methods—responding to Lavater—illustrates the matter well. “[I]n a world in which everything is related through cause and effect,” Lichtenberg writes, science “often places us in a position of inferring from the near to the far, from the visible to the invisible, from the present to the past and future.”^47^ Although the predictive success of classical mechanics was known and celebrated by the late 18th century, Lichtenberg bemoaned the predictive accuracy of meteorology:

But what a vain and wretched piecemeal thing is our science of weather. This is precisely our prophetic art! Despite the volumes of meteorological observations of entire academies, it is still difficult to say in advance whether the sun will shine two days from now. . . . And yet the object of meteorology, as far as I know, is a simple machine whose driving mechanism we will be able to approach more closely in the course of time.^47^

The puzzle is this: if our physical sciences enable us to predict an eclipse with such high accuracy years in advance, how are we unable to predict the weather mere days in advance? Why are classical and celestial mechanics so successful, and meteorology so limited? The answer, Lichtenberg tells us, lies in the respective complexity of the dynamics in question. The magnitude of complexity of factors influencing a human lifespan, however, dwarf both.

[T]he extreme pliability of the body, its perfectibility and corruptibility, whose limits are unknown, comes to the assistance of chance. The wrinkles that form in one person’s skin after a thousand repetitions of the same movement show themselves in another person’s skin after fewer of the same; what causes a distortion and growth on one person that even dogs notice, produces no visible sign on another, at least not as can be detected by the human eye. This shows how pliant everything is, and how a small spark makes the whole go up in flames, while elsewhere it scarcely leaves a scorch mark.^47^

Lichtenberg is here articulating something of what confounds inference to unobservable latent variables in particular natural systems. These are complex systems, which exhibit extreme sensitivity to initial conditions. This is contrasted to the relative ease of inferring latent variables in more simplistic causal dynamics (e.g., the purview of celestial mechanics). The dynamics of our solar system are not chaotic; the weather and human behavior are (although on different orders of magnitude). Thus, if we cannot adequately predict the weather a week in advance, as simplistic a mechanical system it is, relative to the muddled causal mess of a human lifetime, why should we think that unraveling the mysteries of human character and disposition from his outwardly apparent features should be easy? Lichtenberg decries physiognomy as a fool’s errand. Like Galen before him, Lichtenberg critiques physiognomic research on its inability to specify causal mechanisms. Venturing to satirical speculation, he writes: “does the soul fill the body in the manner of an elastic fluid, which always takes the form of the vessel, so that if a flat nose means *schadenfreude*, a man will experience *schadenfreude* if someone presses his nose flat?”^47^ When physiognomists have offered causal theories, these are, by necessity, *ad hoc*: nothing in the mere association of (facial) morphology and enduring character traits relates these mechanistically.

## Epistemic foundations of ML

There is no such thing as philosophy-free science; there is only science whose philosophical baggage is taken on board without examination.

Daniel Dennett^48^

To begin to explain the preponderance of flawed research practices undertaken with ML, we will first delve into the epistemic foundations of ML and contrast these to how it is popularly understood. By epistemic foundations, we mean the basic functionality of these methods, alongside the set of foundational assumptions—sometimes implicit—that drive the overall research and development of ML systems. This discussion requires some digression into issues in philosophy of science and epistemology. Apart from a nascent interdisciplinary field that critically examines algorithmic inference, there is almost no attention paid to the underlying and implicit philosophical problems of statistical ML within the field itself. We examine whether the epistemic foundations of ML, or the meta-narratives surrounding it, make the field particularly vulnerable to pseudoscience.

ML models use evidence, or training data, to form predictions or classifications, which generalize what they have learned from their training set to unseen instances (i.e., novel data). The field of ML strives to automate inductive inference. Thus learning is fundamentally about *generalization*. Many statistical models with algorithmically automated tuning fall under the banner of ML, but it is advances in deep learning that have sparked renewed excitement for the field and that typically underpin the instances of pseudoscientific research practices, which are the target of our critique.

In training a neural network model (DNN), an algorithm tunes the weights of the parameters within the network—called parameterization—in conformity with an objective function, which specifies the desired learning outcome. Through this training procedure, the weights of the network’s parameters come to embody a function mapping inputs to outputs. Assuming certain assumptions hold that guarantee relevant similarities between training and test datasets, such a model is then capable of inferring from observed instances to unobserved instances; from particulars to a more general class.^49^ What is “learned” by the “machine” is hence a mathematical function. The key advantage of these training procedures lies in their ability to discover correlations in very high-dimensional feature spaces.

Classification problems are formulated as the selection of the mathematical function that best fits the mapping of inputs to outputs out of a much broader set of such functions, or hypotheses. The standard supervised ML task trains on a finite sample of labeled examples to make predictions about potentially any input. These models learn functions that map human-defined labels to examples of particular things as represented in data. Though the outputs of ML models are referred to as “predictions,” ML is rarely used to make actual predictions about the future and, despite considerable progress, deep learning still struggles to predict the weather.^50^ In sum, the goal of ML is for an algorithm to train a model to approximate a mathematical function, which can take as its inputs training data and be used to label new examples that were not part of the training sample.^49^

Most ML research focuses on the theoretical problem of learning from example; rather less attention is paid to how the examples from which the model should learn are created. The collection or measurement of data, their subsequent handling and curation, and the construction of categories over which inference is ultimately performed—whether that be in the hand-labeling of data or in the ultimate interpretation of the predictions a model generates—all embody theoretical, and often normative, commitments of human researchers. We argue that a key factor in the spread of computational pseudoscience exists at the level of meta-narratives about science and, in particular, ML-assisted science: those of value- and theory-free induction.

### The value-free ideal in science and ML

Philosophers of science have argued compellingly that scientific practices, and the knowledge they produce, are shaped by human normative values.^51^^,^^52^^,^^53^ Arguments against the so-called value-free ideal locate values at all junctures of the scientific knowledge-production pipeline, including value infiltration of the context of discovery, e.g., determinations of the pursuit-worthiness of various scientific hypotheses or of the representativeness of particular systems relative to the broader class of which they are intended to serve as evidence, and value infiltration of the context of justification (for example, determinations of error tolerance).

On pursuit worthiness, materials discovery, for instance, seeks out molecular structures that support certain engineering applications (e.g., low melting temperature plus high tensile strength). On representativeness, it was historically the case that studies of the male body, e.g., the circulatory or endocrinological systems, were taken to be representative of the *human* circulatory or endocrinological systems. Our understanding of such fundamental biological phenomena as tissue repair meanwhile have been held back by refusal to seriously investigate a most immediate and obvious example: the regular breakdown and rebuilding of the uterine lining over the course of the menstrual cycle in reproductive-age humans with intact ovaries. No doubt the gendered nature of the subject matter has historically led biological and biomedical research programs from deeming menstruation to be either pursuit worthy or representative of biological repair at large.

On error tolerance, many philosophers of science have noted that the considerations of uncertainty quantification and error directionality are, crucially, often value laden. How certain we need to be before we are willing to accept a given hypothesis, e.g., of global temperature change as a function of fossil-fuel burning, depends on both the ultimate dangers presented to humans and the costliness of intervention, not to mention the immediate political ramifications of scientific subjects such as climate. Whether to be more error tolerant for false positives or false negatives further depends on considerations of risk and costliness of erring in either direction—we must be more certain of the presence of tumorous tissues before recommending brain surgery than we must be of bacterial infection before recommending a course of antibiotics.

Most arguments concerning the essential value-ladenness of science center on the underdetermination of our knowledge relative to the results of our empirical efforts (e.g., Douglas^51^). Normative considerations are taken to fill in the evidential gaps. This more foundational epistemologically grounded thesis is overkill for present purposes. Whether or not science, or empirical epistemic pursuits, can, in principle, be rendered value free can be left aside; if an epistemic pursuit is undertaken for the express purpose of direct intervention on human lives, then the epistemic task is ineliminably normatively laden in virtue of its human consequences.

The usage of ML-based predictive and classificatory systems in socially sensitive contexts is thus, necessarily, deeply value laden.^54^ However, the mythology surrounding ML presents it—and justifies its usage in said contexts over the *status quo* of human decision-making—as paradigmatically *objective* in the sense of being free from the influence of human values. This enables the “laundering” of uninterrogated values into the outputs of such ML-based decision-making and decision-support systems, where they are then reified as objective empirical truth.

### The theory-free ideal

Data do not on their own indicate that for which they can serve as evidence.

Helen Longino^53^

A parallel implicit conception of science strives for an even starker vision of objectivity: a science free from theory.^29^ While not so ubiquitous as the value-free ideal, the theory-free ideal has grown since the widespread adoption of domain-generic statistical methods in science. This ideal is now rampant in data-driven fields of research, particularly in the age of ML-assisted science. The *theory* in theory-free ideal is to be understood as any prior commitment or conjecture to the nature of the target system (that is, the phenomenon under study). This is a rational reconstruction of what we take authors to mean when they claim their methods, or the methods of ML at large, to be “free of theory”; see also Andrews.^29^ Much like the value-free ideal, the theory-free ideal comes across as an innocent striving for more objective or more empirical methods; as a meta-narrative, however, it has negative repercussions for science and engineering practice in concealing the necessary input of theory. Here, a scientific meta-narrative is taken to be a tacit, culturally shared background understanding of how science works or ought to work.

The theory-free ideal rarely receives explicit argumentative support; it is most readily seen in the language scientists use to relay their methods and results to one another and the public. Such language describes the methods of science as free from theoretical consideration, free from the bias of human *a priori* judgment, and free from preconceived ontological categories. Perhaps most clearly, one sees the theory-free ideal evidenced in talk of “letting the [raw] data speak for themselves.” Indeed, science popularizers have recently referred to the dawn of data-driven or machine-assisted science as spelling “the end of theory.”^55^

Such a view is underpinned by a widespread belief that the algorithmic discovery of correlations in increasingly large datasets, sans any of the guardrails typical of the practices of trained scientists, is sufficient to count as scientific knowledge. That ML-based studies frequently claim support for specific scientific hypotheses—a sampling of such cases is reviewed in the physiognomy resurrected section—we take as documentation of the existence of such a background belief. Data mining with statistical methods purports to be capable of uncovering new patterns in data not detectable by humans, without the aid of hypothesis or theory. Data, however, are always collected, ordered, deciphered, and interpreted in light of our theories.^56^

Every juncture of the scientific pipeline—and any process that mirrors it in attempting inference from data—is theory inflected. This includes the loci of experimental design, design and calibration of measurement instruments, and methods of quantitative analysis. In the case of ML-based inference, it also includes such steps as problem formulation, model training, model evaluation, and the interpretation and use of the results of an ML modeling procedure.

The necessity of theory is over-determined by such theses as the material theory of induction in philosophy of science, which exposes how empirically established background facts are necessary to license any inductive inference,^57^ and its formal, learning-theoretic equivalent, the no-free-lunch theorems.^58^ The no-free-lunch theorems similarly demonstrate that powerful assumptions about the nature of what is being studied—restrictions on the space of hypotheses—are necessary in order to learn from data.

Further, the data from which ML models “learn” is itself never free of theory but is inexorably “theory-laden.”^59^^,^^60^^,^^61^^,^^62^ Bogen illustrates how it is the very fact that data are not raw, that they are, in a sense, “impure,” that makes them able to serve the meaningful epistemic role they do.^63^ Boyd^59^^,^^64^ argues further that it is not in spite of, but owing to, the theory-ladenness of data that empirical science garners us its epistemic results. For data to serve an evidential role in a scientific inference—and this role is essential to the definition of data—it must hold semantic meaning for epistemic agents to be able to incorporate it into their world knowledge. This comes in the form of being theory laden.

Evaluating the uses of domain-generic statistical methods in the biological, psychological, and social sciences, Richard Lewontin documented hallmarks of a theory-free ideal in the 1970s. Lewontin criticized the misapplication and misinterpretation of such methods as analysis of variance (ANOVA) and principal-component analysis (PCA), alongside linear and multiple regression, revealing the flawed logic of the theory-free ideal and demonstrating how such an approach to empirical inference functions to smuggle hidden assumptions into the final interpretation. The assumption undergirding these statistical malpractices, Lewontin wrote, were that the researchers were “approaching the data in a theory-free manner and that data will ‘speak to them’ through the correlation analysis.”^65^ Further: “Because the methodology of correlation is intrinsically without theoretical content about the real world (that is thought to be its greatest virtue), any statements about the real world must come from the content imported into the analysis.”^65^ The putative objectivity of these methods is often taken to speak in their favor. Real objectivity, of course, or the nearest science is able to get to it, is accomplished by painstakingly unearthing, documenting, and compensating for the many theoretical assumptions, gambits, heuristics, and constraints involved in the work of science.

### Theory and value neutrality in physiognomy

Theory freedom and value freedom appear to have been motivating factors in the specific methodologies chosen by early physiognomists, phrenologists, and eugenicists. Francis Galton, for instance, a physiognomist and the pioneering figure in the eugenics movement, believed that good research practice should consist in “gathering as many facts as possible without any theory or general principle that might prejudice a neutral and objective view of these facts.”^66^ Karl Pearson, a fellow pioneer or both statistical and eugenicist methods, approached research with a similar philosophy: “theorizing about the material basis of heredity or the precise physiological or causal significance of observational results, Pearson argues, will do nothing but damage the progress of the science.”^67^ In collaborative work with Pearson, Weldon emphasized the superiority of data-driven methods that were capable of delivering truths about nature “without introducing any theory.”^68^

Perhaps unsurprisingly, it is precisely this same logic that motivates the usage of ML methods in resurrecting physiognomic research programs in the 21st century. The method of training a model to detect correlations from raw data is justified in being more reliable, objective, or free from (human) bias. It is worth noting that the term “bias” in common language is typically understood to mean something normatively valenced; specifically, something negatively valenced. Biased, in this sense, is effectively synonymous with “discriminatory.” In ML, the term “bias” is often shorthand for “inductive bias,” which refers to the set of assumptions that guide a learning regime to pick up on certain patterns in the data and not others. The no-free-lunch theorems mentioned above tell us that we cannot effectively learn patterns in the absence of such learning biases. Advertising ML modeling techniques as “bias free” is hence best interpreted as signaling both value-freedom and theory-freedom.

Of the studies surveyed in the physiognomy resurrected section, several proclaim their methods to be free of bias, of one form or another. Wu and Zhang,^33^ for instance, present a method for inferring future criminality from still images of human faces, which they claim to be “free of any biases of subjective judgements of human observers.”^33^ In introducing their lie-detection models, Tsuchiya et al.^17^ write that they have “proposed a method to aid in the detection of deception based on machine learning, which excludes human bias from detection.”^17^ Kachur et al.,^9^ who train DNNs to predict latent personality traits from still face images, similarly purport to have “circumvented the reliability limitations of human raters by developing a neural network and training it on a large dataset labelled with self-reported Big Five traits.”^9^

### Potential rebuttal

Some might rebut that, if ML systems are finding robust correlations—robust, at least, enough to generalize—between images of faces and sexual orientation or interview footage and criminality, it *must mean that there is something real* to the relationship between facial features and sexual orientation or gestural tics and criminality. We are not denying that the researchers in the above-outlined instances achieved high classifier accuracy on their targets or that they were able to generalize to holdouts. We are also not denying that achieving high accuracy and limited generalization implicate the presence of robust patterns in the data. What we are questioning is the validity of interpretations lent to these patterns.

That robust patterns exist in natural data should come as no surprise. The world is structured. It contains regularity. All natural data, therefore, contain some measure of regularity. We have to work very hard to produce data containing no learnable regularity. According to a number of mainstream accounts from philosophy of science, the work of science is one of engaging in observation or rigorous measurement procedure that enables us to furnish a causal hypothesis that is explanatory of observed regularity.^69^^,^^70^^,^^71^ There is always regularity imposed on data at multiple places in the process of observation or measurement that generates that data and, further, in preparing it to serve an evidential role in inference. Part of the necessary and difficult work of science is distinguishing between imposed regularity and worldly regularity. There is then the further task of distinguishing between an open-ended number of causal hypotheses that might be explanatory of observed worldly regularity. Again, with natural data, there is *always* both measurement-imposed regularity and objective or worldly regularity. A plurality of hypotheses are always available to explain both. In the case of supervised learning tasks (most real-world applications of ML fall under this heading; all of the studies surveyed in the physiognomy resurrected section are supervised learning tasks), what is being predicted is, straightforwardly, the judgment of the labeler. There is, therefore, no such thing as freedom from human bias in such exercises, and there are myriad ways in which the data come to take on arbitrary features of their collection and labeling conditions.

The trouble with the research studies here surveyed—and physiognomic methods in general—is that these studies present a presumptive (causal) interpretation—whether wittingly or not—of their results without having engaged in the research practices necessary to rule out alternative worldly causal hypotheses, or even ruled out researcher-imposed structure on the data. In the following section, specific errors are highlighted in several recent studies employing physiognomic methods and ML.

Scientists across a number of disciplines, especially the social and psychological sciences, work to establish that their experimental research accords with standards of measurement validity. The framework of measurement validity exists as a guideline for experimental research, ensuring that key concepts are appropriately operationalized and that the results measured are indeed the outcomes of interest. Establishing measurement validity works to guarantee that when we have set out to measure depression we are not merely assessing a transient affective state or that when we seek to establish the positive benefits of hot baths on heart health we have controlled for socioeconomic status, as a recent study failed to do.^72^ None of the studies critically evaluated in this manuscript establish measurement validity (most notably, construct validity). Keystone target-of-prediction concepts are implicitly operationalized in terms of a decision boundary discovered by the trained model, with little or no attention to what is in fact being measured. With such a lax approach to research, the space of confounds that cannot, in principle, be ruled out is effectively open-ended.

## Neo-physiognomy: Exhibit of epistemic failings

Debunking such pseudoscience takes massively more energy than it takes to thoughtlessly produce it.

Iris van Rooij^73^

In the first section of this paper, physiognomy resurrected, we laid out the details of several recent studies whose stated aims were to infer latent human characteristics from records of visual appearances. We briefly recapitulated the results of several such studies, the methods employed, and, in particular, took note of how the data on which models were trained were collected and labeled. In this section, we examine how the methods employed in these studies fail to support the conclusions or capacities alleged by the research authors.

In their 2020 study, Kabir et al.^8^ based their predictions on images “gathered from the web using the web gathering technique,” although the details of this technique were not further elucidated. It is not made clear within the scope of the manuscript on what basis images were classified as normal, drug addicted, autistic, or criminal. However, it seems highly probable, given the exemplars presented, given the lack of methodology cited in their classification of the four categories, and given their description of their dataset sourcing, that the researchers built their dataset from web data clustered according to query terms. This is to say, the researchers collated their data from datasets openly available on the web by prompting with autism, criminal, drug addict, etc., and their classifications were hence solely based on the results of search engine queries. There are a number of problems with this method. Google image search, for instance, when queried with the terms “autism,” or “autistic person,” produces images largely of individuals with Down syndrome or other chromosomal disorders. According to our best modern medical knowledge, there are no expressed morphological differences in autistic individuals.^74^^,^^75^ From the small sample set displayed in the paper, it is clear that 100% of the subjects in the autism category have chromosomal disorders. Chromosomal disorders are roughly as frequent in patients with autism spectrum disorder (ASD) as in the general population; certain chromosomal disorders carry a moderately increased risk of ASD. If a classifier is trained on images of individuals with chromosomal disorders labeled as autistic, it will learn whatever visually perceptible phenotypic variations cluster with chromosomal disorders. Similarly, when one queries a search engine for images of convicted criminals, one retrieves—for reasons that should strike the reader as obvious—almost exclusively mugshots. Very few images will be staged images for magazines or for media, etc. These images, importantly, typically replicate either the settings or the facial expressions typical of mugshots. Crucially, obvious confounds that are readily picked up by a classifier are clearly present for all the labels Kabir et al.^8^ set out to predict.

Wu and Zhang^33^ claim that their methods are able to infer “criminality based solely on still face images, which is free of any biases of subjective judgments of human observers.”^33^ To begin, the concept of criminal propensity is a human construct, and the data, to the extent that they encode this concept, do so only by virtue of having been shaped by human judgment of criminality. There is no objective signal of criminality that can be discovered independently of human judgment. “At the onset of this study” the researchers assumed that the “modern tools of machine learning and computer vision” would “refute the validity of physiognomy, although the outcomes turn out otherwise.”^33^ In other words, Wu and Zhang^33^ take their achievement of high classifier accuracy (89.51% for convnets) to be a validation of physiognomic methods. To repeat, physiognomy is the logic of inferring psychological or behavioral dispositions on the basis of the appearance of physical features, such as facial features, on the supposition that these traits are correlated owing to a common biological cause. Wu and Zhang^33^ examine the relation between innate traits and social behaviors on the one hand, and physical characteristics on the other, claiming that one would be hard pressed to find “a more convincing” validation of this relation “than examining the success rates of discriminating between criminals and non-criminals with modern automatic classifiers.”^33^ The researchers relied on monochrome photographs of human faces from government-issued IDs, all males residing in mainland China. These methods were proclaimed to control for confounds and over-fitting.

However, with a dataset of fewer than 2,000 images in total, it is impossible to control for over-fitting. By relying on ID photographs, the only factors controlled for were those specific to camera, lighting conditions, and at least in principle, the positioning of the head, factors that, e.g., Kabir et al.^8^ and Wang and Kosinski^20^,^21^ do not control for. The failing point of this approach is that there remain an open-ended number of possible confounds. Simply discovering a correlation between photographs of faces and a “convicted of crime” label cannot lend support for the conclusion of an underlying biological mechanism. Only mechanistic biological evidence would suffice to lend credence to the hypothesis of a shared mechanism, for only mechanistic biological evidence would avoid most of the myriad confounding factors possible in such an inference. For instance, it is plausible that individuals who have been convicted of a crime would show in facial posture the hallmarks of unhappier affect relative to individuals who have not been convicted of a crime. Further, features of attire and grooming reliably track socioeconomic status, which correlates heavily with likelihood of arrest and conviction (a statistic that differs notably from crime rates). For reasons of selection bias, among others, convicted criminals may also vary systematically by age. Indeed, although Wu and Zhang^33^ only reveal three images corresponding to each label in their training set, all criminals appeared to wear black t-shirts and to be of an advanced age and dour affect, while the law-abiding citizens all wore white-collared shirts and suit jackets, were younger, and smiled. Nevertheless, Wu and Zhang^33^ claim their study to have produced “strong empirical evidence for the validity of automated face-induced inference on criminality.” In stark contrast to this bold claim of success, in a subsequent response to critics, the researchers disparage interlocutors who interpreted the original study as supporting the idea that such methods might actually function in practice, accusing them of succumbing to the base rate fallacy.^76^

Wang and Kosinski^20^,^21^ sought to infer sexual orientation from still face images. The researchers scraped social media profiles for front-facing photographs of subjects and inferred the hidden label of sexual orientation (gay or straight) from further scraped datapoints, including social media group memberships and “likes.” Again, the authors alleged that their results provided “strong support for the PHT, which argues that same-gender sexual orientation stems from the underexposure of male fetuses and overexposure of female fetuses to prenatal androgens responsible for the sexual differentiation of faces, preferences, and behavior.”^20^,^21^ While editorial intervention changed this phrasing to portray that the study furnished results “consistent with PHT [*sic*]” in the final, published version of the manuscript, the intent of the study, along with its takeaways and potential use cases, remain unaltered. The claim is thus that the achievement of high classifier accuracy on a discrimination task between homosexual and heterosexual subjects in photographs constituted powerful scientific evidence for a specific proposed biological mechanism undergirding sexual orientation. The hypothesis is that atypical exposure to testosterone in the womb results in both the “deviant” sexuality of same-sex attraction and atypical facial morphology. Homosexual males are thus suggested to have abnormally effeminate facial structure relative to their heterosexual counterparts. In homosexual women, the reverse is proposed to be the case. Wang and Kosinski^20^,^21^ contended that their results strongly lend credence to this theory. The idea that prenatal hormone exposure plays an exclusive or even dominant role in the determination of adult sexual preference is inconsistent with modern biological evidence.^77^

Wang and Kosinski^20^,^21^ supplemented their homosexuality classifier with a further task, which purported to measure the degree to which the homosexual face deviated from gender-typical facial morphology. The reasoning here, however, is clearly seen to be circular: the gender-prototypical facial morphology is pre-defined by the same measures as utilized in the original sexuality classification task. “Deviation” therefrom in the case of homosexual facial morphology is hence a given, so long as a decision boundary can be drawn between heterosexual and homosexual faces, as the first part of the study affirms. Wang and Kosinski^20^,^21^ claim that these results demonstrate over- or underexposure to testosterone in the womb to be the biological mechanism leading to both gender-atypical facial morphology and homosexual tendencies. The discovery that DNNs for feature extraction in tandem with regression techniques are apparently capable of discriminating homosexual and heterosexual faces from self-taken photographs, however, cannot serve as evidence for a specific biological mechanism. Only controlled, experimental, biological evidence can lend support to a specific mechanistic hypothesis. Otherwise, a plethora of potential confounds can never be ruled out. The confounds, in the case of this study, turned out to be, in the first place, grooming choices: in particular, the wearing of glasses versus contacts, the presence of absence of makeup, and the presence or absence of facial hair. The most powerful signal, however, came from head tilt and angle from which the photo was taken.^26^

Wang and Kosinski took the overwhelming critical response to their 2018 paper to heart. In a 2024 study co-authored with Poruz Khambatta, the researchers sought to construct an experiment capable of predicting latent identities—in this instance, political leanings—from facial images, this time controlling for many of the potential confounds that critics had gestured to.^10^ The resultant network exhibited weak accuracy in predicting political alignment (reported by the researchers in the form of Pearson product-moment correlation, with a value of *r* = 0.22; product moment scores range from −1 to 1, with 0.0 being null correlation). Heatmaps of predictive salience were generated to elucidate what features of the images were most highly determinative of classifier output. The researchers further produced composite images of study participants and averaged the facial proportions of the liberal and conservative clusters to produce a visual demonstration of the features that may have been critical in predicting political orientation.

The slant of the eyebrows, the nostrils, the curl of the outer lip, and the outer edge of the jaw were revealed from the saliency map to have been almost exclusively determinative of predictive outcome. As y Arcas et al.^26^ note, it is precisely these four loci on the face that are subject to the strongest delta in proportion to the relative forward tilt of the head. The narrative depicted by the heatmap was confirmed by composite image and facial outlines: “The average facial outlines . . . suggest that liberals had smaller lower faces. This is also visible on the average faces. . . . Note that liberals’ lips and noses are shifted downward, and their chins are smaller.”^10^ The hypothesis that liberal-leaning individuals are biologically disposed to large foreheads, narrow chins, inward slanted brows, and small mouths appears to be the one favored by the authors. A much more parsimonious explanation holds that all of these features are indicative of a forward tilt of the head. Indeed, individuals who self-identified as conservative leaning exhibited the reverse pattern on the composite photograph: wider relative jaw width, upward-slanted eyebrows, more visible nostrils, and the inverse inflection of the outer corners of the mouth. Although Kosinski et al.^10^ went to great pains to control for confounds, including grooming choices, posture, and expression, these measures appear to have been insufficient to keep the research subjects’ heads in place. While the college student participants were instructed to ensure that the “chin is at a 90-degree angle to [the] body,” it might be ventured that the average 19-year-old falls short of a perfectly calibrated proprioceptive sensibility of 90° chin-to-body angle.

Although Kosinski et al.^10^ were less bold in their assuredness of biological mechanism than in their homosexuality research, they nevertheless suggested “endocrinological, genetic, [or] developmental factors” underpinning the apparent association between face and political orientation, re-affirming their belief in the validity of physiognomic methods. We recommend that, in future investigations, Kosinski et al. might consider a well-known experimental device in cognitive and neurosciences: the bite bar. This device secures research participants’ jaws and keeps head tilt from being a confounding factor. It has the added benefit of ensuring uniformity in the expression of the mouth. The addition of this precaution, however, would still not be sufficient to rule out the majority of potential confounds, and would not make the inference from classifier accuracy to biological mechanism any more secure.

## The vulnerability of ML to pseudoscience

There is no greater impediment to progress in the sciences than the desire to see it take place too quickly.

Georg Christoph Lichtenberg

The use of ML-based systems to infer behavioral traits, clinical conditions, interior states, or life outcomes on the basis of images recapitulates the essential inferential logic of physiognomy in eras past. In spite of the manner in which these projects are advertised, nothing about the involvement of ML in the task puts the inference on more solid epistemic footing. We believe that a variety of factors contribute to the resurgence of physiognomic research practices in the field of ML.

In the first place, any attempt at inductive inference about some system in the world on the basis of data collected from that system is a kind of scientific inference or, at least, mirrors the fundamental structure of scientific inference and can be thought of as a science-adjacent task. Thus, any machine-assisted attempt to infer latent unobservable phenomena from human appearances, even in industrial application, is attempted science, or a mimicry thereof. Typically, those who carry out scientific inference are scientists trained exhaustively in the protocols of a particular scientific paradigm and inculcated with a wealth of domain knowledge.

Trained scientists possess an abundance of information concerning what is known and unknown in relation to their subject matter, which investigatory avenues have borne fruit, and which have been dead ends. Those inducted into a scientific research tradition have access, also, to a wealth of cautionary tales: they have learned from not only the wisdom but the naivety of generations of scientists before them. While neither omniscient nor infallible, scientists are, in virtue of their requisite disciplinary training, aware of what is considered to be established knowledge in their respective fields within some bounds of uncertainty and aware of what is considered to have been debunked or refuted.

When embarking on a project in applied ML, it is not standard practice to read the historical legacy of domain-specific research. For any applied ML project, there exists a field or fields of research devoted to the study of that subject matter, be it on housing markets or human emotions. This ahistoricity contributes to a lack of understanding of the subject matter and of the evolution of methods with which it has been studied. The wealth of both subject-matter expertise and methodological training possessed by trained scientists is typically not known to ML developers and practitioners.

The gatekeeping methods present in scientific disciplines that typically prevent pseudoscientific research practices from getting through are not present for applied ML in either industry or academic research settings. The same lack of domain expertise and subject-matter-specific methodological training characteristic of those undertaking applied ML projects is typically also lacking in corporate oversight mechanisms as well as among reviewers at generalist ML conferences. ML has largely shrugged off the yoke of traditional peer-review mechanisms, opting instead to disseminate research via online archive platforms. ML scholars do not submit their work to refereed academic journals. Research in ML receives visibility and acclaim when it is accepted for presentation at a prestigious conference. However, it is typically shared and cited, and its methods built upon and extended, without first having gone through a peer-review process. This changes the function of refereeing scholarship. The peer-review process that does exist for ML conferences does not exist for the purpose of selecting which work is suitable for public consumption but, rather, as a kind of merit-awarding mechanism. The process awards (the appearance of) novelty and clear quantitative results. Even relative to the modified functional role of refereeing in ML, however, peer-reviewing procedures in the field are widely acknowledged to be ineffective and unprincipled.^78^,^79^ Reviewers are often overburdened and ill-equipped to the task. What is more, they are neither trained nor incentivized to review fairly or to prioritize meaningful measures of success and adequacy in the work they are reviewing.

This brings us to the matter of perverse incentives in ML engineering and scholarship. Both ML qua academic field and ML qua software engineering profession possess a culture that pushes to maximize output and quantitative gains at the cost of appropriate training and quality control. In most scientific domains, a student is not standardly expected to publish until the PhD, at which point they have typically had at least half a decade of training in the field. Within ML, it is now typical for students to have their names on several papers upon exiting their undergraduate. The incentives force scholars and scholars in training to churn out ever higher quantities of research. As limited biological agents, however, there is a bottleneck on time and critical thought that can be devoted to research. As quantity of output is pushed ever higher, the quality of scholarship necessarily degrades.

The field of ML has a culture of obsession with quantification—a kind of “measurement mania.” Determinations of success or failure at every stage and level are made quantitatively. Quantitative measures are intrinsically limited in how informative they can be—they are, as we have said, only informative to the extent that they are lent content by a theory or narrative. Quantitative measure cannot, for instance, capture the relative soundness of problem formulation. It has been widely acknowledged that benchmarking is given undue import in the field of ML and, in many cases, is actively harmful in that it penalizes careful theorizing while rewarding kludgy or hardware-based solutions.^78^

A further contributing factor is the increased distribution of labor within scientific and science-adjacent activities. The Taylorization or industrialization of science and engineering pushes its practitioners into increasingly specialized roles whose operations are increasingly opaque to one another. This fact is not intrinsically negative—its repercussions for the legitimacy of science can be, when care is taken, a net positive. In combination with the other facets already mentioned, however, it can cause a host of problems. Increasingly, scholars and industry actors outsource the collection and labeling of their data to third parties. When—as we have argued—much of the theoretical commitments of a modeling exercise come in at the level of data collection and labeling, offloading these tasks can have damaging repercussions for the epistemic integrity of research.

All of the above realities work alongside a basic fact of modern ML: its ease of use. With data in hand and the computing power necessary to train a model, it is possible to achieve publishable or actionable results with a few hours of scripting and write-up. The rapidity with which such models are able to be trained and deployed works alongside a lack of gatekeeping and critical oversight to ill effect.

## What is the harm in failed inference?

ML models are increasingly utilized across all walks of life, from particle physics to finance, while ML-based automated decision-making and decision-support systems increasingly dictate or inform life-altering outcomes for citizens. The use of ML or its products in the designing of real-world interventions or the architecting of public-facing technologies should, in general, be understood as cementing causal interpretations of the outputs of such models. Acting on model outputs is *de facto* causal interpretation. Misinterpretation of the causal mechanisms responsible for observed data in socially sensitive contexts should be expected to harm classified populations. Some illustrations should make this intuitive.

Say that a medical AI system is inputted with the symptoms a patient presents with on intake: nausea, vomiting, unusual sweating, and tightness in the chest, alongside neck, jaw, and upper back pain. The system advises that the patient is likely experiencing a bout of anxiety; they are issued a prescription for benzodiazepines and sent home. Doctors have hence acted in accordance with the theory that a psychiatric condition caused the presentation of symptoms observed in the patient. The symptoms described, however, are typical symptoms of heart attack in women. Most studies of heart attack presentation have rested on observations of male patients. Women are therefore 50% more likely to go undiagnosed for myocardial infarction as their male counterparts (and twice as likely to have symptoms of heart attack misdiagnosed as psychiatric).^80^ If the medical ML system in question has been trained on physician diagnostic histories and the usual slough of medical studies without explicit corrections for demographics, it can be expected that the classifier will inherit the biases of its training data. The outcome is that our female heart attack patient is sent home untreated, endangering her life.

Take a second example: a bank’s lending algorithm disproportionately recommends denying loans to black applicants. Research has shown that such classifiers, even when blinded to the race label, have sufficient proxy data to faithfully reconstruct race. The bank acts on this recommendation in its approval or denial of loan applications. In doing so, the bank reifies the hypothesis that something in the disposition or circumstances of the individual loan applicant makes them, in reality, less likely to pay that loan. It rules out the competing hypotheses that the bank has historically (arbitrarily) denied loans to black individuals or that it differentially sets loan repayment rates for different sub-populations. The result, at the individual level, is that a loan-worthy applicant is denied a loan. At the collective level, the result is that banking practices continue to withhold prospects for wealth accumulation, home ownership, and upward mobility from black communities.

The recent flurry of studies that have leveraged deep-learning methods to predict enduring personality, identity, or behavioral features from human likenesses have proposed applications for their methods in surveillance,^8^ law enforcement,^7^^,^^17^^,^^33^ the identification of military targets,^22^ early prediction of mental illness,^8^^,^^16^ detection of academic dishonesty,^17^^,^^24^ and hiring,^17^^,^^33^ among others. Potential harms flowing from the misdiagnosis of a causal hypothesis in these applications include false detainment and imprisonment, extermination of innocent civilians, false diagnosis, exclusion from educational opportunities, and exclusion from employment opportunities. This list is not intended to be exhaustive but is merely suggestive.

Many pathways by which epistemic failures lead to harms are more convoluted than the above. If, for instance, as Wang and Kosinski^20^,^21^ claim their results suggest, *in utero* androgen exposure were determinative of sexuality in adulthood, homosexuality might be medically induced or prevented. Needless to say, if such a thing were possible, this would have drastic bio-ethical implications alongside immediate implications for the gay community. However, we know this not to be the case. Already in the 1990s, it was established that prenatal hormonal factors alone could not be responsible for the determination of sexual preference. Twin studies revealed that “52 percent of identical twin brothers of gay men also were gay, compared with 22 percent of fraternal twins, compared with 11 percent of genetically unrelated brothers.”^81^ Clearly, the endocrinological environment of the maternal womb cannot be the sole causal factor determining sexual orientation, according to the methods of human genetics.

It has further been pointed out that automatic gender recognition (AGR) techniques, a sub-field of facial recognition that aims to algorithmically identify the gender of individuals from photographs or videos, might pose grave threats to gender non-conforming people.^82^ The building of homosexuality-identification software has been decried widely as irresponsible given the present global political landscape.^83^,^84^ As of 2024, 63 countries outlaw homosexuality; of these, 11 nations punish homosexuality with the death penalty.^85^ The confabulation of results via pseudoscientific methods can exact immeasurable harms on already marginalized communities. Authoritarian governments already actively use such technologies to suppress dissent and repress human rights.^86^

## Discussion

In the course of this manuscript, we have surveyed a recent deluge of ML-enabled neo-physiognomic research techniques. We have examined in detail the methodological—and hence, epistemological— deficiencies of these studies. Matters of the ethicality of deployed ML/AI systems, we argue, are rarely, in practice, separable from their epistemic failings. When ML-based systems are deployed or their results acted upon in real-world, socially sensitive contexts, their results are lent a *de facto* causal interpretation. When an intervention or technology is predicated on a misconstrual of the causal structure of the system acted upon, the results of said intervention or technology are predisposed toward being both harmful and unpredictable. For these reasons, we urge that the fields of ML ethics and responsible AI prioritize the following:

(1) Increased awareness of functionality issues and their intersection with ethical concerns, à la Raji et al.^3^
(2) The debunking of popular misconceptions about the epistemic status of ML systems, à la Andrews^29^ and Johnson.^54^
(3) The promotion of subject-matter-appropriate research standards and guardrails to enforce them, including project oversight mechanisms and more adept reviewing practices, as advocated by, e.g., Lipton and Steinhardt,^78^ Sloane and Moss,^87^ and Jacobs.^88^
(4) The development of pedagogical resources detailing research best practices, à la Kapoor et al.^89^ and Guest.^90^
(5) Historical awareness of research traditions in which applied ML projects are, wittingly or not, participating, à la Harvey et al.,^91^ alongside increased interdisciplinary collaboration with scholars trained in those research traditions.
(6) Awareness of the complex web of incentive structures within which all research will inevitably be interpreted and acted upon, which may pervert the outcomes of even well-intentioned work if it rests on shaky epistemic foundations.

The last of these is important for ensuring that research carried out in an ethically conscientious spirit is not developed in harmful directions by forces outside of the researchers’ control. Many of the authors of the studies surveyed in this paper have urged that they do not intend the tools built to ever be used. In the case of Wang and Kosinski,^20^,^21^ the authors even claim that their work is intended to serve as a warning against the deployment of such a tool. However, opportunistic firms have, predictably, seized at the opportunity to capitalize on what they take to be newly revealed engineering capabilities. Awareness of the limitations of regulatory measures and the incentives governing industry practitioners must play a role in decisions concerning whether to pursue or to publicize particular research avenues.
